# Strigolactones Modulate Salicylic Acid-Mediated Disease Resistance in *Arabidopsis thaliana*

**DOI:** 10.3390/ijms23095246

**Published:** 2022-05-08

**Authors:** Miyuki Kusajima, Moeka Fujita, Khamsalath Soudthedlath, Hidemitsu Nakamura, Koichi Yoneyama, Takahito Nomura, Kohki Akiyama, Akiko Maruyama-Nakashita, Tadao Asami, Hideo Nakashita

**Affiliations:** 1 Graduate School of Agricultural and Life Sciences, The University of Tokyo, Tokyo 113-8657, Japan; kusajima@g.ecc.u-tokyo.ac.jp (M.K.); ahide87@g.ecc.u-tokyo.ac.jp (H.N.); asami@g.ecc.u-tokyo.ac.jp (T.A.); 2 Department of Bioscience and Biotechnology, Fukui Prefectural University, Fukui 910-1195, Japan; pt-fujita@fpu.ac.jp; 3 Graduate School of Bioresource and Bioenvironmental Sciences, Kyushu University, Fukuoka 819-0395, Japan; khamsalath.soudthedlath.682@s.kyushu-u.ac.jp (K.S.); amaru@agr.kyushu-u.ac.jp (A.M.-N.); 4 Center for Bioscience Research and Education, Utsunomiya University, Tochigi 321-8505, Japan; yone2000@sirius.ocn.ne.jp (K.Y.); tnomura@cc.utsunomiya-u.ac.jp (T.N.); 5 Graduate School of Life and Environmental Sciences, Osaka Prefecture University, Osaka 599-8531, Japan; akiyama@biochem.osakafu-u.ac.jp

**Keywords:** Arabidopsis, disease, phytohormones, strigolactones, salicylic acid, systemic acquired resistance, GR24, strigolactone biosynthesis inhibitor, ethylene

## Abstract

Strigolactones are low-molecular-weight phytohormones that play several roles in plants, such as regulation of shoot branching and interactions with arbuscular mycorrhizal fungi and parasitic weeds. Recently, strigolactones have been shown to be involved in plant responses to abiotic and biotic stress conditions. Herein, we analyzed the effects of strigolactones on systemic acquired resistance induced through salicylic acid-mediated signaling. We observed that the systemic acquired resistance inducer enhanced disease resistance in strigolactone-signaling and biosynthesis-deficient mutants. However, the amount of endogenous salicylic acid and the expression levels of salicylic acid-responsive genes were lower in strigolactone signaling-deficient *max2* mutants than in wildtype plants. In both the wildtype and strigolactone biosynthesis-deficient mutants, the strigolactone analog GR24 enhanced disease resistance, whereas treatment with a strigolactone biosynthesis inhibitor suppressed disease resistance in the wildtype. Before inoculation of wildtype plants with pathogenic bacteria, treatment with GR24 did not induce defense-related genes; however, salicylic acid-responsive defense genes were rapidly induced after pathogenic infection. These findings suggest that strigolactones have a priming effect on *Arabidopsis thaliana* by inducing salicylic acid-mediated disease resistance.

## 1. Introduction

Plants are exposed to a range of biotic and abiotic stress factors, such as pathogen attack, insect herbivory, and environmental stress. To adapt to such adverse conditions, plants have evolved unique defense mechanisms that are regulated by phytohormones such as salicylic acid (SA), jasmonic acid (JA), ethylene (ET), and abscisic acid (ABA). These phytohormones contribute to biotic stress responses through a complex network involving synergistic and antagonistic interactions, although SA- and JA/ET-mediated defense responses are important for the resistance against biotrophic and necrotrophic pathogens, respectively [1,2,3,4,5,6].

In plant immunity, pathogen-associated molecular patterns (PAMPs) are first recognized by receptors on the surface of plant cells, activating a basal resistance called PAMP-triggered immunity (PTI). Virulent pathogens have developed effector proteins that suppress this PTI mechanism of host plants; however, resistant plants have acquired a strong immune mechanism, called effector-triggered immunity (ETI), which overcomes pathogenic effectors [7]. Systemic acquired resistance (SAR), induced by SA, is a potent innate immunity system in plants that is effective against a wide range of pathogens [8]. The induction of SAR in infected plants is accompanied by SA accumulation through the increased expression of SA synthase, *ISOCHORISMATE SYNTHASE 1* (*ICS1*), and pathogenesis-related (*PR*) genes [9]. Further, a reduction of the SA-receptor protein NPR1 is reportedly important for SAR induction, to which an increase in total glutathione (GSH) level contributes [10]. Additionally, probenazole and its derivative, 1, 2-benzisothiazol-3(2*H*)-one-1, 1-dioxide (BIT), induce SAR by activating its signaling pathway upstream of SA [11,12]. Furthermore, several studies have reported SAR as an effective strategy for disease control in plants [11,12,13,14,15,16], and priming of phytohormone-mediated disease resistance has been reported as a different type of plant immunity system. Although only weak or no expression at all of the major defense-related genes through SA- or JA-mediated signaling pathways are observed in primed plants by interaction with arbuscular mycorrhizal (AM) fungi [17,18], nonpathogenic bacteria [19,20], or treatment with certain chemicals [21], they can still respond more rapidly and strongly to pathogenic infection to protect themselves.

Strigolactones (SLs) are carotenoid-derived metabolites that act as germination stimulants on root parasitic weeds of the genus *Striga* [22]. Furthermore, SLs secreted by host plants induce mycelial branching in rhizosphere AM fungi and stimulate symbiosis [23]. Recently, SLs have been reported to inhibit shoot branching [24,25]. Thus, SLs have been categorized as a novel class of phytohormones.

Analysis using carotenoid-deficient mutants and carotenoid biosynthesis inhibitors suggests that SLs are synthesized from carotenoids [26]. Moreover, several mutants, including *Arabidopsis more axillary growth* (*max*) exhibiting increased shoot branching, have been identified as SL biosynthesis mutants. Arabidopsis *max3* and *max4* mutants have mutations in *CAROTENOID CLEAVAGE DIOXYGENASE 7* (*CCD7*) and *CCD8*, respectively, and their phenotypes are restored upon treatment with synthetic SLs, indicating that they are SL-deficient mutants [25]. Additionally, AtD27, which exhibits β-carotene isomerase activity and converts all-trans-β-carotenes, is reportedly involved in SL biosynthesis. Furthermore, carlactone (CL), an SL biosynthetic intermediate, is synthesized in plants from all-trans-β-carotene using three different enzymes [27]. Mutant *max1* is deficient in cytochrome P450 oxygenase CYP711A, which converts CL into carlactonic acid in the SL biosynthetic pathway [28]. Similarly, the shoot-branching mutant, SL-insensitive *max2*, lacks the F-box protein that plays a role in recognizing SLs [29]. In addition, the SL-insensitive mutant *d14* has a mutation in the gene encoding an α/β-hydrolyzing enzyme. After binding with SL, the SL receptor D14 interacts with SCF (SKP1-CUL1-F-box) containing the F-box protein MAX2, which transmits SL-mediated signals by degrading target proteins through the ubiquitin–proteasome system [30]. The biological activities of SLs have been analyzed using SL-related mutants and bioactive chemicals. Shoot branching is inhibited by the synthetic SL-analog GR24, and it is improved by the SL-biosynthesis inhibitor TIS108, which inhibits CYP711A activity [31].

SLs are also involved in plant responses to environmental stress, as in drought [32,33], salt [34], and cold [35] resistance. As SLs are carotenoid derivatives that alleviate environmental stress as ABA does, interactions between SL and ABA have been proposed. Thus, for example, Arabidopsis SL biosynthesis-deficient (*max3* and *max4*) and SL signaling-deficient (*max2*) mutants exhibit low drought tolerance and are less sensitive to ABA [36]. However, some reports indicate that Arabidopsis *max2* is less drought-tolerant and more ABA-sensitive, whereas *max3* and *max4* are as tolerant as the wildtype (WT) [37]. Altogether, these studies suggest that *MAX2*-mediated SL signaling interacts with ABA signaling during stress tolerance.

Furthermore, recent studies suggest the specific role of SLs in plant responses to biotic stress: Tomato SL biosynthesis-deficient mutant *ccd8* exhibits increased susceptibility to *Botrytis cinerea* and *Alternaria alternata* [38]. Similarly, rice SL signaling-deficient mutant *d14* and SL biosynthesis-deficient mutant *d17* are susceptible to *Magnaporthe oryzae* [39]. Further, Arabidopsis *max2*, *max3*, *max4*, and *d14* mutants are highly susceptible to the biotrophic virulent bacterial pathogen *Pseudomonas syringae* pv. *tomato* DC3000 (*Pst*) [40,41]. The model compatible-interaction system between Arabidopsis and *Pst*, in which the SA-mediated defense signaling is activated upon infection, has contributed to clarifying the detailed mechanisms at work behind the plant immune system [42]. However, the mechanism underlying the effects of SLs on SA-mediated disease resistance remains unclear. Therefore, to gain a better understanding of the role of SL in disease resistance, the aim of this study is to determine the effects of SLs on SA-mediated defense responses in Arabidopsis upon *Pst* infection.

## 2. Results

### 2.1. Relationship between SLs and SA Signaling Pathway in Arabidopsis

To evaluate the effects of SLs on SAR induction, we examined SAR induction in the SL biosynthesis-deficient mutants *max1*, *max3*, and *max4* and in the SL signaling-deficient mutant *max2*. SAR was induced by BIT applied using soil drench treatment to avoid its dilution during dip-inoculation with the pathogen. Furthermore, to avoid the influence of stomatal aperture on disease resistance, we covered plants with plastic wrap and maintained high humidity for 12 h before and after pathogen inoculation.

**Figure 1:**
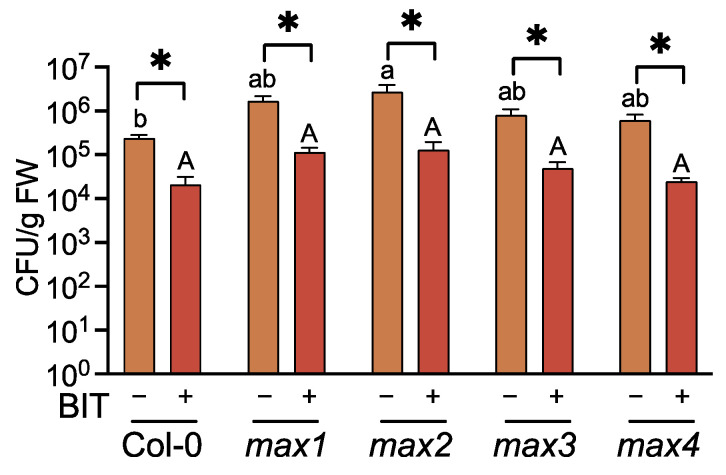
Effects of BIT treatment on growth of *Pseudomonas syringae* pv. *tomato* DC3000 in Arabidopsis leaf tissues. Three-week-old plants were treated with water (control) (–) or BIT (1 mg/pot) (+) using the soil drenching method 3 d before inoculation with *Pst* (2 × 10^5^ CFU/mL). Leaves were homogenized at 3 d post-inoculation, and the number of CFU was estimated using their growth on nutrient broth agar plates. Values are means ± SE from six replicates, each with three leaves. Significant differences between the control and BIT-treated leaves are indicated by * (unpaired *t*-test, *p* < 0.05). Different letters indicate significant differences between accessions of the same treatment (Tukey’s multiple comparison test, *p* < 0.05).

**Figure 2:**
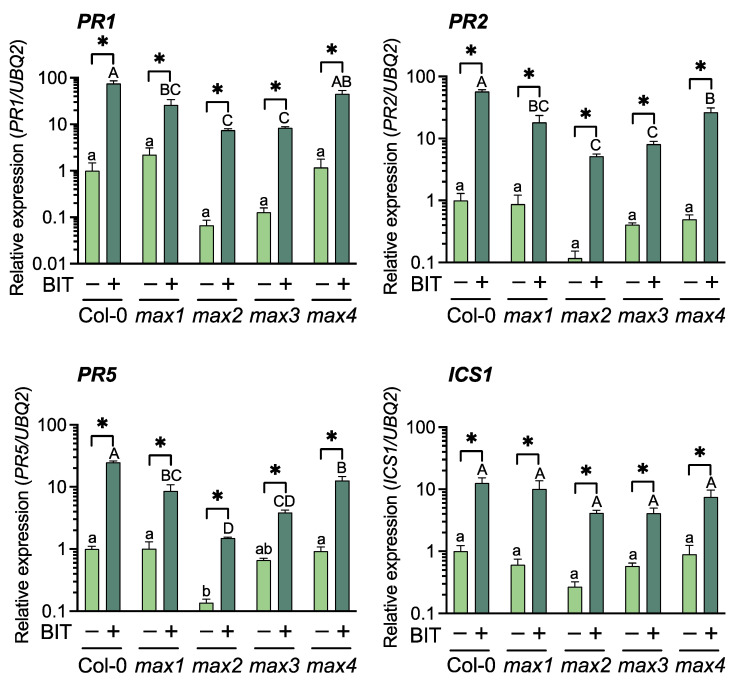
Expression of defense-related genes in BIT-treated plants. Leaves of three-week-old plants were sprayed with water (control) (–) or 1 mM BIT (+) and collected after 3 d. Transcript levels were normalized using the expression of *UBQ2* (At2g36170) in the same samples. Relative mRNA levels among treatments are presented. Values are means ± SE (*n* = 3). Significant differences between the control and BIT-treated plants are indicated by * (unpaired *t*-test, *p* < 0.05). Different letters indicate significant differences between accessions of the same treatment (Tukey’s multiple comparison test, *p* < 0.05).

**Figure 3:**
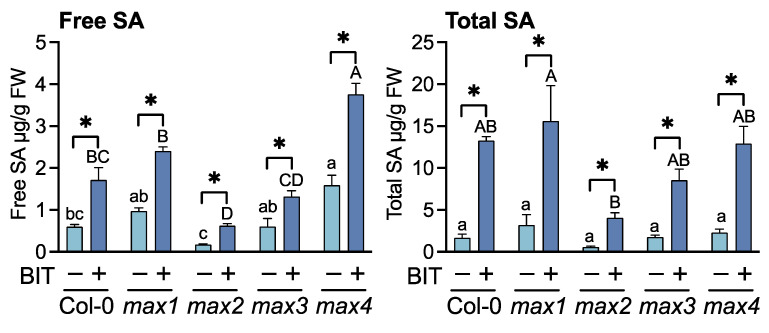
Accumulation of free and total SA in BIT-treated plants. Plants were treated with water (control) (–) or 1 mM BIT (+). Leaves were harvested 3 d after treatment, and free and total SA (free SA + SA glucoside) levels were quantified using high-performance liquid chromatography. Significant differences between control and BIT-treated plants are indicated by * (unpaired *t*-test, *p* < 0.05). Different letters indicate significant differences between accessions of the same treatment (Tukey’s multiple comparison test, *p* < 0.05).

The effects of BIT treatment on SAR induction in the SL-related mutants were evaluated using a pathogenic bacterial inoculation test, defense gene expression profiling, and the estimation of endogenous SA content. Resistance to *Pst* was assessed by measuring bacterial growth in the leaf tissues. At 3 d after inoculation, leaves of all the SL-related mutants treated with BIT exhibited > 10-fold lower bacterial titers than those of the untreated (control) plants as well as WT (Col-0) plants (Figure 1). Reverse transcription-quantitative polymerase chain reaction (RT-qPCR) indicated that upon treatment with BIT, the expression levels of *PR1* (At2g14610), *PR2* (At3g57260), and *PR5* (At1g75040) were upregulated in the SL-related mutants and the WT, compared to the control plants (Figure 2). Moreover, BIT treatment significantly upregulated *ICS1* (At1g74710) expression and increased the levels of free and total endogenous SA (free SA + SA glucoside) in the SL-related mutants and in the WT (Figure 2 and Figure 3). These findings suggest that SAR can be induced in SL biosynthesis- and signaling-deficient mutants.

In the absence of SAR induction, bacterial growth on the leaf tissues was significantly higher in the mutant *max2* than in WT plants, whereas bacterial growth in mutants *max1*, *max3*, and *max4* was intermediate between those of *max2* and WT (Figure 1). This finding suggested that SL-mediated signaling is partially involved in basal resistance against *Pst*. Although *ICS1* levels in BIT-treated plants did not differ between the SL-related mutants and the WT, SA levels were altered among these plants, with *max2* and *max4* exhibiting the lowest and highest levels of both free and total SA, respectively (Figure 2 and Figure 3).

Consistent with SA levels, the levels of expression of *PR* genes after BIT treatment were significantly lower in *max2* than in WT (Figure 2). Thus, BIT-mediated activation of SA biosynthesis and signaling pathways were weaker in *max2* than in WT, which can be associated with high bacterial titer in the leaves of *max2* (Figure 1). Additionally, the level of activation of the SA-mediated signaling pathway and disease resistance in other SL-related mutants was intermediate between those of *max2* and WT (Figure 1 and Figure 2). These findings suggest that the absence of SL signaling affected the SA-mediated signaling pathway and, consequently, the degree of disease resistance against *Pst*. Furthermore, the difference between SL biosynthesis- and signaling-deficient mutants can be explained by the fact that the inhibition of SL-mediated signaling was stronger in the mutant *max2*, which is defective in the F-box protein MAX2, than in other biosynthesis-deficient mutants.

### 2.2. Effects of the Synthetic SL Analog GR24 on SA-Mediated Disease Resistance

**Figure 4:**
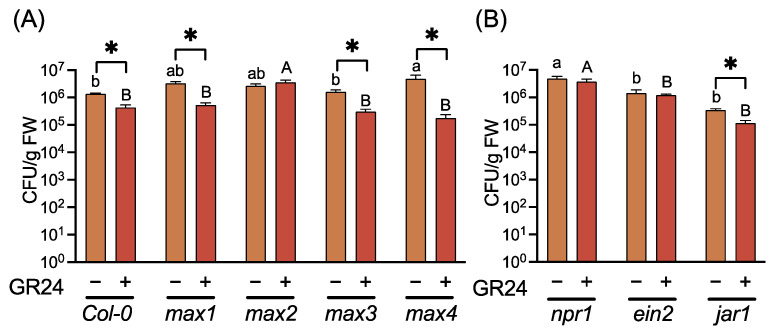
Effects of GR24 treatment on growth of *Pseudomonas syringae* pv. *tomato* DC3000 in Arabidopsis leaf tissues. Three-week-old plants of WT and SL-related mutants (**A**) and SA-, ET-, and JA-related mutants (**B**) were treated with 0.02% acetone (control) (–) or 10 µM GR24 (+) by drenching the soil 3 d before inoculation with *Pst* (2 × 10^5^ CFU/mL). Leaves were homogenized 3 d post-inoculation, and the number of CFUs was estimated from their growth on nutrient broth agar plates. Values are means ± SE of six replicates, each with three leaves. Significant differences between control and GR24-treated plants are indicated by * (unpaired *t*-test, *p* < 0.05). Different letters indicate significant differences between accessions of the same treatment (Tukey’s multiple comparison test, *p* < 0.05).

To reveal the effects of SLs on SA-mediated disease resistance, we examined the effects of GR24, a synthetic SL analog, on resistance against *Pst* in Arabidopsis. Treatment of WT plants with GR24 improved their resistance against *Pst* (Figure 4). Furthermore, GR24-treated SLs biosynthesis-deficient mutants (*max1*, *max3*, and *max4*) exhibited reduced bacterial growth in the leaf tissues compared to the untreated control plants. In contrast, GR24 treatment did not affect disease resistance in *max2* (Figure 4). These results indicate that the activation of SL-mediated signaling by GR24 improved disease resistance in Arabidopsis.

To determine the role of disease resistance-related phytohormones in GR24-induced disease resistance, induction of resistance against *Pst* was analyzed in SA-, JA-, and ET-mediated signaling defective mutants *npr1*, *jar1*, and *ein2,* respectively. GR24 treatment induced disease resistance only in *jar1*, indicating that SA and ET—but not JA—were involved in GR24-induced disease resistance (Figure 4).

**Figure 5:**
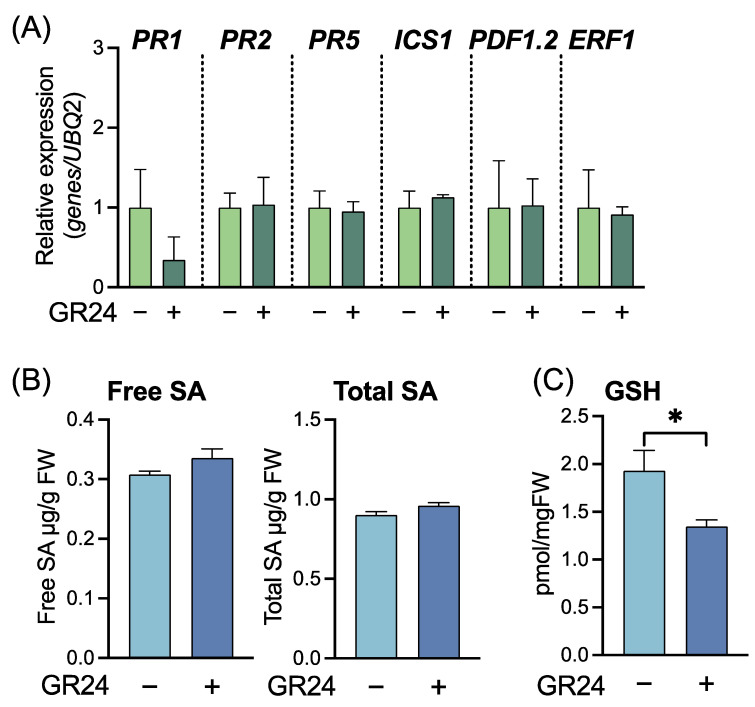
Analysis of defense-related gene expression and metabolite accumulation in *Arabidopsis thaliana* treated with GR24. Three-week-old plants were treated with 0.02% acetone (control) (–) or 10 µM GR24 (+) using the soil drenching method, and leaves were collected 3 d after treatment. (**A**) Expression levels of *PR1*, *PR2*, *PR5*, *ICS1*, *PDF1.2*, and *ERF1* in GR24-treated plants. Transcript levels were normalized using the expression of *UBQ2* in the same samples. Relative mRNA levels of each gene among the treatments are presented. Values are means ± SE (*n* = 4). (**B**,**C**) Accumulation of free and total SA and GSH in GR24-treated plants. The levels of free and total SA (free SA + SA glucoside) and GSH were quantified using high-performance liquid chromatography. Significant differences between control and GR24-treated plants are indicated by * (unpaired *t*-test, *p* < 0.05).

To characterize GR24-induced disease resistance, we examined physiological changes in the GR24-treated WT plants. The level of expression of SAR marker genes, namely *PR1*, *PR2*, and *PR5*, and *ICS1* did not increase in GR24-treated plants, indicating that the mechanism of GR24-induced disease resistance was different from that of SAR (Figure 5A). Furthermore, the expression levels of *PDF1.2* (At5g44420), the JA-related gene, and *ERF1* (At4g17500), the ET-related gene, were not significantly different between GR24-treated and untreated control plants (Figure 5A), indicating that JA- and ET-mediated signaling pathways were not activated in GR24-treated plants. Endogenous accumulation of SAR-related metabolites was examined three days after GR24 treatment. The levels of free SA and total SA (free SA + SA glucoside) were not influenced by GR24 treatment (Figure 5B). Although an increase in GSH level is important for SA-mediated signaling in SAR induction, the level of GSH in GR24-treated plants was slightly but significantly lower than that of untreated control plants. These findings suggest that the mechanism underlying GR24-induced disease resistance was independent of SA-, JA-, or ET-mediated signaling pathways.

**Figure 6:**
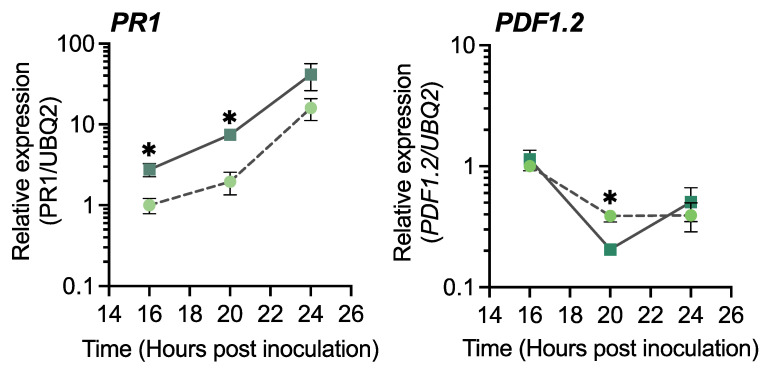
Expression of *PR1* and *PDF1.2* after pathogen infection in GR24-treated plants. Three-week-old plants were treated with 0.02% acetone (control) or 10 µM GR24 by drenching the soil 3 d before inoculation with *Pst* (2 × 10^7^ CFU/mL). Leaves were collected 16, 20, and 24 h after *Pst* inoculation. Closed circle, water-treated control plants; closed square, GR24-treated plants. Significant differences between control and GR24 treatments are indicated by * (unpaired *t*-test, *p* < 0.05).

To determine the mechanism underlying GR24-induced resistance against *Pst*, defense responses were investigated by analyzing gene expression upon pathogen inoculation. Time-course analysis indicated that *PR1* was significantly upregulated in GR24-treated plants, compared with control plants, at 16 and 20 h post inoculation, whereas similar levels were maintained up to 24 h (Figure 6). This finding suggests that the defense system was rapidly activated in the GR24-treated plants, which should contribute to the increased resistance against *Pst*. Conversely, the expression of *PDF1.2* after *Pst* infection was not upregulated but downregulated due to activation of SA-mediated signaling (Figure 6). Therefore, GR24-induced disease resistance showed a priming effect that activated major defense pathways, such as the SA-mediated defense pathway, after infection.

### 2.3. Effects of the SL Biosynthesis Inhibitor TIS108 on Disease Resistance

Pathogen inoculation assays and gene expression analyses indicated that GR24 primed plants against *Pst* infection, suggesting that SLs positively regulated disease resistance. This was further supported by the fact that *in-planta Pst* growth was higher in *max* mutants than in WT plants (Figure 1). However, *max* mutants, with the absence of SLs from germination, may have some side effects on the stress response systems during their growth.

**Figure 7:**
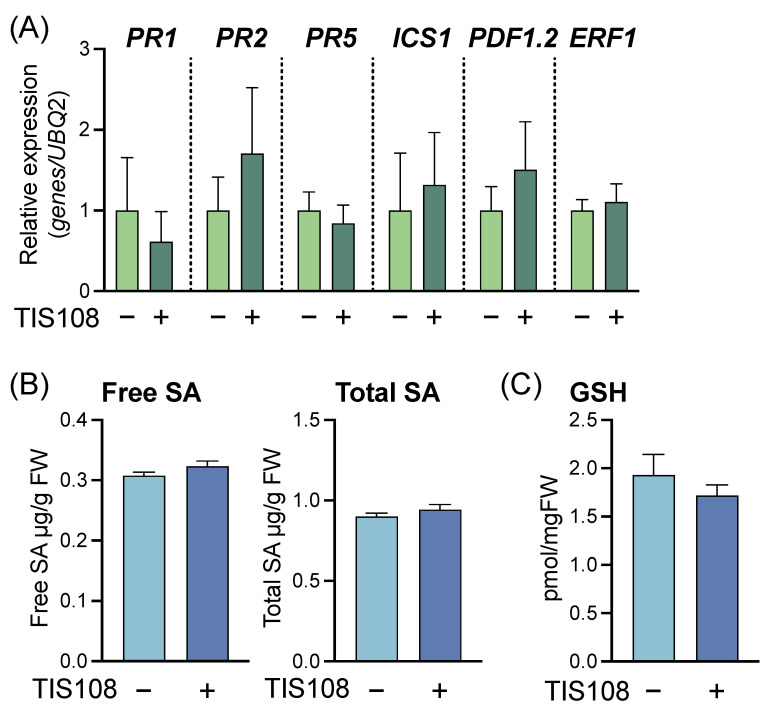
Analysis of defense-related gene expression and metabolite accumulation in *Arabidopsis thaliana* treated with TIS108. Three-week-old plants were treated with 0,02% dimethyl sulfoxide (DMSO) (–) or 10 µM TIS108 (+) using the soil drenching method, and leaves were collected 3 d after treatment. (**A**) Expression levels of *PR1*, *PR2*, *PR5*, *ICS1*, *PDF1.2*, and *ERF1* in TIS108-treated plants. Transcript levels were normalized using the expression of *UBQ2* in the same samples. Relative mRNA levels of each gene among the treatments are presented. Values are means ± SE (*n* = 4). (**B**,**C**) Accumulation of free and total SA and GSH in TIS108-treated plants. The levels of free and total SA (free SA + SA glucoside) and GSH were quantified using high-performance liquid chromatography.

**Figure 8:**
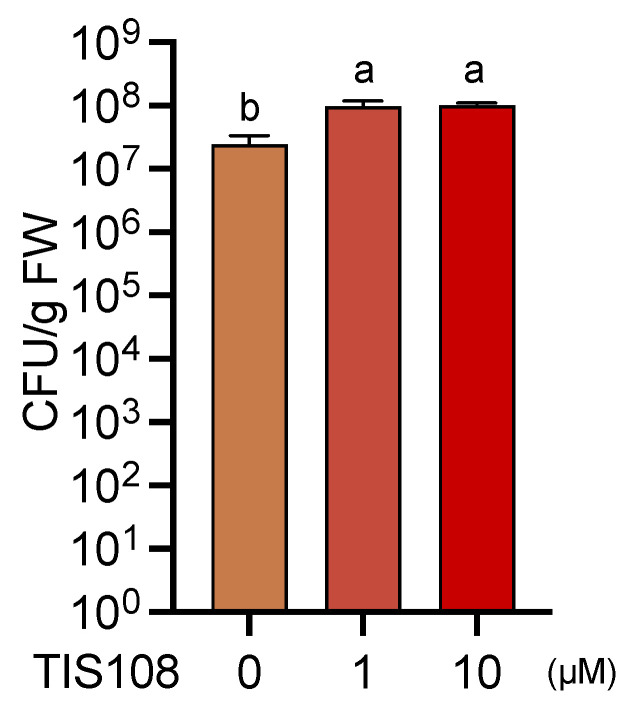
Effects of strigolactone biosynthesis inhibitor TIS108 on *Pst* growth in Arabidopsis leaf tissues. Three-week-old plants were treated with 0.02% dimethyl sulfoxide (DMSO) (control) or 1 or 10 µM TIS108 by drenching the soil 3 d before inoculation with *Pst* (2 × 10^5^ CFU/mL). Leaves were homogenized 3 d post-inoculation, and the number of CFUs was estimated using their growth on nutrient broth agar plates. Values are means ± SE of six replicates, each with three leaves. Different letters indicate significant differences (Tukey’s multiple comparison test, *p* < 0.05).

To verify the contribution of endogenous SLs to the resistance against *Pst*, we examined the effects of the SL-biosynthesis inhibitor TIS108 on gene expression, metabolite accumulation, and disease resistance. The expression levels of SAR-related genes *PR1*, *PR2*, *PR5,* and *ICS1*; JA-related gene *PDF1.2*; and ET-related gene *ERF1* were not affected by TIS108 treatment (Figure 7A). Endogenous levels of free SA, total SA, and GSH were not significantly different between the TIS108-treated and untreated control plants (Figure 7B). Treatment with as low as 1 µM TIS108 significantly decreased disease resistance in WT plants compared to that of the untreated control plants, suggesting that endogenous SLs contribute to *Pst* resistance (Figure 8).

## 3. Discussion

**Figure 9:**
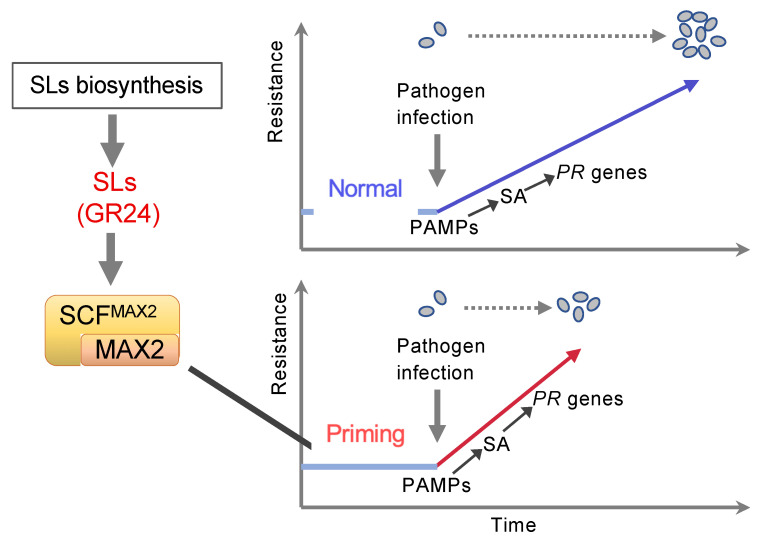
Proposed model for modulation mechanism of SA-mediated defense signaling by strigolactones. SCF^MAX2^ is SKP1-CUL1-F-box complex containing MAX2 as F-box protein.

Various aspects of plant immunity are regulated by phytohormones such as SA, JA, and ET. Previous studies have demonstrated the role of SLs in disease resistance using SLs-biosynthesis and signaling mutants of rice and Arabidopsis [39,40]. This study demonstrated that SLs positively regulated SA-mediated signaling during basal defense responses but not SAR induction. Increased disease resistance upon GR24 treatment proved to be a case of priming of the plant immune system, that is, a rapidly upregulated expression of defense genes upon infection (Figure 6). Moreover, the reduction in disease resistance upon treatment with the SL biosynthesis-inhibitor, TIS108, strongly supported that SL-mediated signaling positively affected SA-mediated disease resistance (Figure 8). These findings suggest that SLs play a positive role in plant immunity, especially in the SA-mediated disease resistance. Similar rapid upregulation of defense-related gene expression was observed in plants primed by symbioses with microbes or chemicals [18,19,21]. In turn, this study is the first demonstration of a priming effect on plants by exogenous and endogenous SLs; however, how SLs contribute to plant disease resistance in nature remains to be elucidated. Although whether SLs-biosynthesis can be activated by pathogen attack is unknown, SLs probably affect some unidentified mechanism along the pathway between PAMPs recognition and expression of defense-related genes, such as SA-biosynthesis-related genes and *PR* genes, which enables primed plants to counterattack pathogens more rapidly than unprimed plants (Figure 9).

As SLs comprise an important class of phytohormones with various roles in plant growth and development, there may be some side effects on the physiology of SL-related mutants due to the absence of SLs. Similarly, TIS108 treatment should also have some side effects, as it suppressed SL-mediated signaling. However, such effects did not occur in the short-time treatment with TIS108, which was supported by our finding that the expression of defense-related genes and the accumulation of SA were not affected by TIS108 treatment.

In Arabidopsis SL biosynthesis- and signaling-defective mutants, as well as in WT plants, a SAR inducer improved disease resistance by activating SA biosynthesis and SA-mediated signaling, suggesting that SLs do not affect SAR induction (Figure 1). Further, and consistently with a previous report [40], without SAR induction, these mutants were more susceptible to *Pst* than WT plants (Figure 1), and, compared to other SL-related mutants, the SL-signal-perception-defective mutant *max2* exhibited a different phenotype. Furthermore, regardless of SAR induction, the disease resistance and SA-mediated signaling processes, such as *PR* gene expression and SA accumulation, were suppressed in *max2* compared to WT plants (Figure 1). In contrast, SL biosynthesis-related mutants exhibited such processes at levels that were intermediate between those observed for *max2* mutant and WT plants (Figure 1). These findings suggest that SLs are important, at least for SA-mediated disease resistance.

When the pathogen was inoculated via foliar spray, the levels of disease resistance were lower in *max2* than in the other SLs biosynthesis-deficient mutants, which was attributed to the increased number of open stomata in the *max2* mutant [40]. In this study, to avoid the influence of stomatal opening and closing, pathogen inoculation was performed by dipping the plants into the bacterial suspension after keeping 100% RH for 12 h before and after inoculation. Thus, the differences in disease resistance and SA-mediated signaling did not result from differences in stomatal opening (Figure 1). We hypothesize that the mutations in MAX2 affected SL-mediated signaling by altering SL-signal perception, thereby affecting the SA-mediated disease resistance. In this case, the differences in SA-mediated signaling processes among SL-biosynthesis-defective mutants would likely be owing to the different physiological effects of each specific mutation, a plausible idea that warrants further research.

Treatment with GR24 failed to induce disease resistance in the mutant *npr1,* indicating that an SA signal was involved in SL-mediated disease resistance (Figure 4), which is consistent with the altered SA-mediated disease resistance in SL-related mutants (Figure 1). However, SAR induction via the activation of SA-mediated signaling was observed in SL-related mutants, suggesting that SLs are not directly involved in SA-mediated disease resistance. Alternatively, a plausible mechanism involves the regulation of SA-mediated signaling by SL during basal resistance, including PTI. However, further investigation is necessary to confirm the operation of this mechanism.

Treatment with GR24 effectively induced disease resistance in *jar1* but not in *ein2*, indicating that ET but not JA was involved in SL-mediated disease resistance (Figure 4). Further, some reports indicate that ET is involved in SL-mediated signaling; thus, for example, SL-induced seed germination in *Striga hermonthica* (Delile) Benth. requires ET biosynthesis and signaling [43]. Additionally, SL promotes ET accumulation in etiolated Arabidopsis seedlings by upregulating the expression of 1-aminocyclopropane-1-carboxylic acid oxidase (ACO) [44]. Similarly, SL promotes ET biosynthesis in Arabidopsis roots [45]. In this study, ET-responsive *ERF1* was not induced in GR24-treated plant leaves (Figure 5), suggesting that ET was not involved in SL-mediated priming.

In contrast, several other reports have underlined the importance of ET in SA-mediated disease resistance; however, such findings remain controversial. In Arabidopsis, co-treatment with an ET biosynthetic precursor (1-aminocyclopropane-1-carboxylic acid) and SA upregulated *PR1* relative to SA treatment alone [46]. Conversely, *ein3 eil1*, a double mutant of ET-responsive transcription factors, constantly accumulated SA, indicating that ET negatively affects SA-mediated signaling pathways [47]. Nonetheless, GR24-induced disease resistance required ET-mediated signaling through *EIN2* (Figure 5), suggesting that ET improved SA-mediated disease resistance after pathogen infection of primed plants.

Nutrient stress, such as low phosphate or nitrogen, induces SL biosynthesis, which alters SL-mediated responses in AM fungi symbioses, which effectively increase the soil volume explored by plant roots in search for phosphorus. However, the trade-off between growth and disease resistance is well known. In contrast to plants growing under soil nutrient-rich conditions (i.e., excess phosphate or nitrogen for better growth), which are more susceptible to diseases, plants growing under soil nutrient-poor conditions exhibit higher levels of expression of defense-related genes after infection [48]. Overall, SL synthesized under soil nutrient-poor conditions may not only promote symbiosis with AM fungi but effectively prime plants for infection as well. This effect might be possible because priming does not affect growth, while it effectively protects plants from pathogen attack by accelerating the induction of SA-, JA-, or other phytohormone-mediated disease resistance mechanisms.

## 4. Materials and Methods

### 4.1. Plant Material and Chemical Treatment

*Arabidopsis thaliana* Col-0 and seven mutants with the same ecotype, including *max1-1* [24], *max2-1*, *max3-1*, *max4-1*, *npr1-2*, *jar1-1,* and *ein2-1*, were used for the experiments [9]. Plants were grown in sterilized potting soil (Nihon hiryo, Fujioka, Japan) in plastic pots (5 cm × 5 cm × 5 cm) inside a growth chamber under a 16:8 h light: dark photoperiod at 22 °C and 60% RH.

BIT [11] solutions (1 mg/mL for soil drenching and 1 mM for spraying) were prepared by dissolving the BIT powder in sterilized distilled water. GR24 (rac-GR24) and TIS108 [31] solutions (1 mM) were prepared from 50 mM GR24 in acetone and 50 mM TIS108 in DMSO, respectively, by diluting with sterilized distilled water. Three-week-old plants were treated with chemicals as indicated in the Figures.

### 4.2. Pathogenicity Assays

Control and chemical-treated plants were inoculated with *Pst* as previously described [49].

### 4.3. Estimation of SA Levels

Three-week-old plants were treated with chemicals for three days. Leaf samples (ca. 100 mg) were harvested and used for extraction and measurement of SA as previously described [12]. Briefly, leaf tissues were homogenized in 1 mL of 90% methanol and then extracted using 1 mL of 100% methanol. These extracts were pooled and dried at 40 °C. The dried residues were extracted in 4 mL of water at 80 °C for 15 min, and equal aliquots (1 mL) were used for free and total SA estimation. One aliquot was extracted using 2.5 mL ethyl acetate–cyclohexane (1:1) following the addition of 50 µL of concentrated HCl, and the upper layer was dried and dissolved in 1 mL of 20% methanol in 20 mM sodium acetate buffer (pH 5). This solution was then subjected to high-performance liquid chromatography (HPLC) analysis to determine free SA levels. Then, 1 mL of β-glucosidase (Merck, Darmstadt, Germany) solution (3 units/mL) was added to the remaining aliquots and incubated for 6 h. These aliquots were prepared for HPLC analysis using the above procedure to determine total SA levels. SA analysis was performed using an Agilent 1220 Infinity LC (Agilent, Snata Clara, CA, USA) equipped with a ZORBAX Eclipse Plus C18 (4.6 × 150 mm; Agilent) and run with 20% methanol in 20 mM sodium acetate buffer (pH 5) at a flow rate of 1 mL/min. SA was detected and fluorometrically quantified at 295 nm excitation and 370 nm emission wavelengths.

### 4.4. GSH Measurement

Three-week-old plants were treated with chemicals for three days. Leaf samples (ca. 100 mg) were harvested and used for extraction and measurement of GSH as previously described [50].

### 4.5. RT-qPCR

Gene expression profiles were determined using leaf samples. Total RNA isolation, cDNA synthesis, and RT-qPCR were performed as previously described [49]. Gene-specific primers used in this study are listed in Supplementary Table S1. Transcript levels were normalized using the expression of *UBQ2* in the same samples [51].

## Data Availability

Data are available on request.

## Supplementary Materials

The following supporting information can be downloaded at: https://www.mdpi.com/article/10.3390/ijms23095246/s1.
